# Crawling the German Health Web: Exploratory Study and Graph Analysis

**DOI:** 10.2196/17853

**Published:** 2020-07-24

**Authors:** Richard Zowalla, Thomas Wetter, Daniel Pfeifer

**Affiliations:** 1 Department of Medical Informatics Heilbronn University Heilbronn Germany; 2 Center for Machine Learning Heilbronn University Heilbronn Germany; 3 Institute of Medical Biometry and Informatics Heidelberg University Heidelberg Germany; 4 Institute of Software and Information Systems Engineering Ben-Gurion University of the Negev Beer Sheva Israel; 5 Department of Biomedical Informatics and Medical Education University of Washington Seattle, WA United States

**Keywords:** health information, internet, web crawling, distributed system

## Abstract

**Background:**

The internet has become an increasingly important resource for health information. However, with a growing amount of web pages, it is nearly impossible for humans to manually keep track of evolving and continuously changing content in the health domain. To better understand the nature of all web-based health information as given in a specific language, it is important to identify (1) information hubs for the health domain, (2) content providers of high prestige, and (3) important topics and trends in the health-related web. In this context, an automatic web crawling approach can provide the necessary data for a computational and statistical analysis to answer (1) to (3).

**Objective:**

This study demonstrates the suitability of a focused crawler for the acquisition of the German Health Web (GHW) which includes all health-related web content of the three mostly German speaking countries Germany, Austria and Switzerland. Based on the gathered data, we provide a preliminary analysis of the GHW’s graph structure covering its size, most important content providers and a ratio of public to private stakeholders. In addition, we provide our experiences in building and operating such a highly scalable crawler.

**Methods:**

A support vector machine classifier was trained on a large data set acquired from various German content providers to distinguish between health-related and non–health-related web pages. The classifier was evaluated using accuracy, recall and precision on an 80/20 training/test split (TD1) and against a crowd-validated data set (TD2). To implement the crawler, we extended the open-source framework StormCrawler. The actual crawl was conducted for 227 days. The crawler was evaluated by using harvest rate and its recall was estimated using a seed-target approach.

**Results:**

In total, n=22,405 seed URLs with country-code top level domains .de: 85.36% (19,126/22,405), .at: 6.83% (1530/22,405), .ch: 7.81% (1749/22,405), were collected from Curlie and a previous crawl. The text classifier achieved an accuracy on TD1 of 0.937 (TD2=0.966), a precision on TD1 of 0.934 (TD2=0.954) and a recall on TD1 of 0.944 (TD2=0.989). The crawl yields 13.5 million presumably relevant and 119.5 million nonrelevant web pages. The average harvest rate was 19.76%; recall was 0.821 (4105/5000 targets found). The resulting host-aggregated graph contains 215,372 nodes and 403,175 edges (network diameter=25; average path length=6.466; average degree=1.872; average in-degree=1.892; average out-degree=1.845; modularity=0.723). Among the 25 top-ranked pages for each country (according to PageRank), 40% (30/75) were web sites published by public institutions. 25% (19/75) were published by nonprofit organizations and 35% (26/75) by private organizations or individuals.

**Conclusions:**

The results indicate, that the presented crawler is a suitable method for acquiring a large fraction of the GHW. As desired, the computed statistical data allows for determining major information hubs and important content providers on the GHW. In the future, the acquired data may be used to assess important topics and trends but also to build health-specific search engines.

## Introduction

### Overview

The internet has become an increasingly important resource for health information, especially for laypeople [1-10]. Web users perform online searches to obtain health information regarding diseases, diagnoses, and different treatments [1]. However, with a growing amount of web pages, it is nearly impossible for humans to manually keep track of evolving and continuously changing content in the health domain. According to the (German) Good Practice Guidelines for Health Information, “evidence-based health information is...a trustworthy state of the medical knowledge” [11]. Even if health information is found via well-known search engines, it does not necessarily meet with this definition and may be influenced by commercial interests [12].

Therefore, it is important to identify health content providers and assess their relevance [13]. In this context, an automatic web crawling approach can help to understand the structure of the health-related web (ie all web pages offering health-related information). By focusing only on such content, it is possible to (1) identify information hubs for the health domain, (2) find content providers of high prestige, and (3) identify important topics and trends within the health-related web. In future work, the identified content providers of high prestige could be analyzed for their respective trustworthiness and their compliance with the criteria of evidence-based health information [11].

According to Van der Bosch et al [14], in 2015 the (indexed) web was estimated to consist of roughly 47 billion web pages. However, only a fraction of those web pages contain health-related information. So, in order to determine the structure of the health-related web, it is crucial to determine for each web page’s content whether it is health-related or not.

A related filter method can be used within a web crawler to filter out irrelevant web pages, therefore reducing the total number of web pages that need to be crawled. This saves time and financial resources for the crawling task. Nevertheless, analyzing such an amount of data requires high performance hardware and parallelization approaches.

Yet, to the best of the authors’ knowledge, no study has been previously conducted and published about the health-related web. This study provides a first analysis of the health-related web limited to web pages in German, the so-called German health web (GHW). In this regard, we restrict our study to the three mostly German-speaking countries Germany, Austria and Switzerland (D-A-CH).

A distributed focused crawler for the GHW is outlined and evaluated as part of this study. Using the acquired data it is possible to extract the graph structure of the GHW for the goals listed above and provide access to health-related text material for linguistic analysis and further research purposes.

### Related Work

#### Importance of Health Information on the Web

The World Wide Web and its graph structure have been a subject of study for many years [15-17]. However, domain-specific and/or country-dependent analysis of graph properties have not been the primary scope of research in the recent years [18,19]. Moreover, a review by Kumar et al [20] shows that research related to focused crawling was popular in the late 1990s and mid 2000s but seems to have lost attention in the last decade. As the internet is an important resource for health information [21], finding relevant content remains an important task [8].

#### Web Crawling of Health Information

In 2005, Tang et al [22] investigated the use of focused crawling techniques to assess the topic relevance and quality of medical information. For this purpose, n=160 seeds from the category depression of the Open Directory Project (now Curlie) were selected. They found that such an approach fetches twice as many pages as a crawler without topic focus. In another study, Pirkola et al [23] described the use of focused crawlers to acquire text from the genomics domain. They found, that “the source of seed URLs and the issues related to the multilinguality of the web” are major challenges in this area.

Abbasi et al [24] used a focused crawler to collect credible medical sentiments and opinions in the context of drug surveillance. In this context, their crawler was evaluated on “a set of 100 seed URLs pertaining to health and drug-related websites” and achieved a harvest rate of 10.06% (1,243,074/12,362,406). In 2016, Abbasi et al [25] demonstrated the use of a focused crawler to acquire credible online medical content in the context of postmarket drug surveillance. Their method was able to “collect over 80% of all relevant credible content in the first 20% of the crawl.”

In Xu et al [26], a user-oriented adaptive focused crawler was implemented and applied in the cancer domain (ie, on breast and lung cancer). The authors found “that the new crawler can substantially accelerate the online user-generated content acquisition efforts for cancer researchers.”

Amalia et al [27] presented a focused crawler for the acquisition of health-related articles written in Indonesian. In this study, different crawling strategies and their relative impacts on crawler performance were investigated. They found that crawling larger sites first improves the number of crawled articles.

In 2016, Rheinländer et al [28] studied the scalability of an information extraction framework using a focused crawling approach to collect and analyze “a 1 TB collection of web text from the biomedical domain” written in English. For this purpose, they generated a set of n=485,462 seeds using commercial search engines with which their focused crawler achieved a harvest rate of 38%.

### Aims of the Study

The authors decided to concentrate on health-related web pages available free of charge on the internet in the D-A-CH region that can be found under the respective country-code top-level domains (ccTLDs) .de, .at, and .ch. In this context, the aim of this study was fourfold:

- Demonstrate the suitability of a focused crawler approach for the acquisition of health-related content in the D-A-CH region
- Provide a curated list of seed points for the health domain in the D-A-CH region
- Provide a crowd-validated evaluation data set consisting of health-related and non–health-related URLs that can be used to evaluate other classifiers used in focused crawlers for the health domain in the D-A-CH region
- Give preliminary insights into the graph structure of the GHW

To the best of the authors’ knowledge, no similar study has been previously conducted on a large scale. In particular, this has not been done for the GHW.

Besides a statistical analysis of the GHW, this paper shares our experience in building and operating a highly scalable focused crawler. Thus, researchers who want to perform a similar analysis for web pages of the health domain in their country can benefit from the experiences gained.

## Methods

### Focused Web Crawling

#### Basic Web Crawling Process

**Figure 1:**
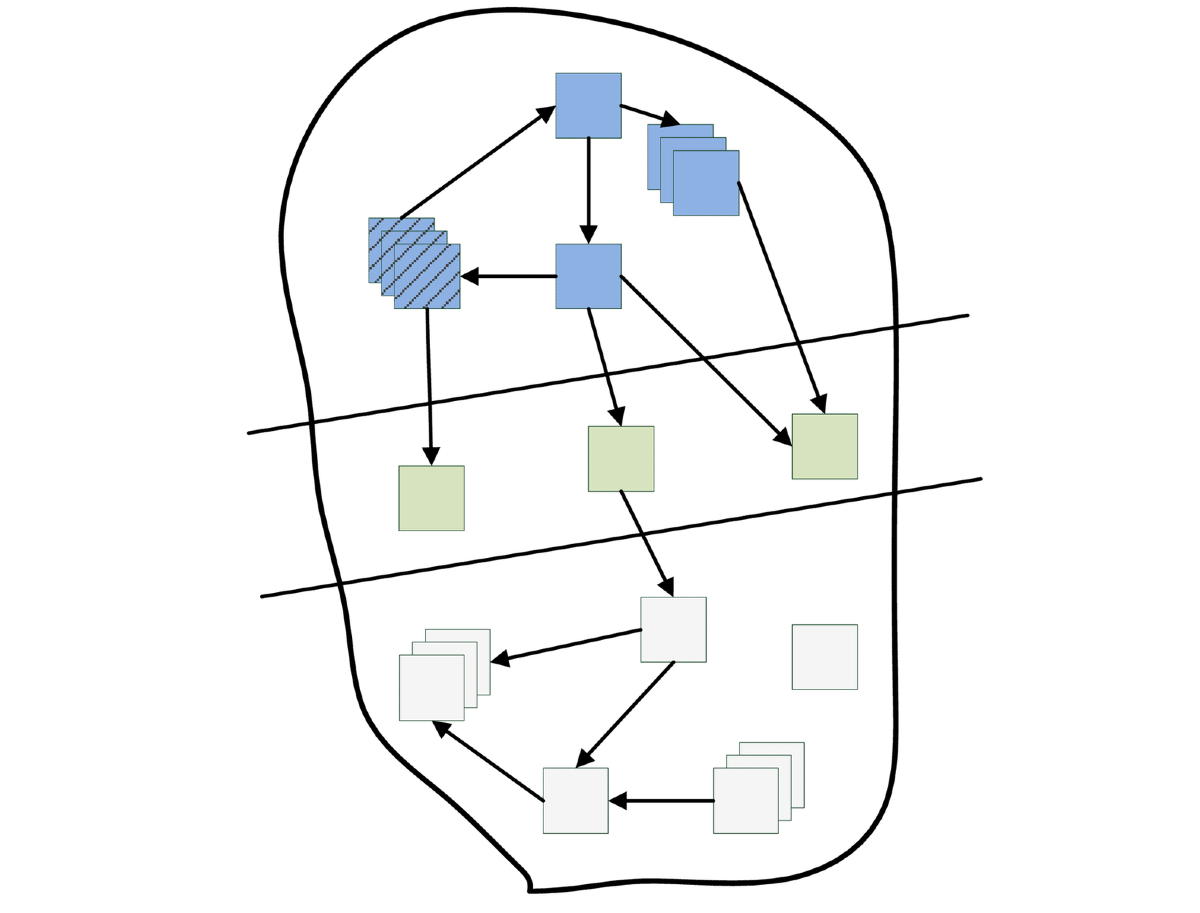
Schematic representation of the web graph traversal by a crawler. Pages colored in blue represent processed pages; in green, pages referenced in the frontier; in gray, undiscovered web content. Pages in dashed blue represent so-called initial seed pages.

As depicted in Figure 1, a web crawler traverses the directed graph of the web [29,30]. Starting from a given set of seed URLs, the web crawler fetches web pages. After the download is successful, the HTML of a web page is parsed and hyperlinks to other web pages are extracted. These links are then analyzed and added in a priority queue called frontier [30,31]. The web graph is then visited via those URLs kept in the frontier. The crawler repeats this process until the frontier is empty or it is stopped manually.

Due to the enormous size of the web [14], one must focus on a certain domain of interest to speed up the crawl. In this context, a focused crawler only visits those outgoing links of a web site that appear to be relevant for the given topic. To determine whether a link is relevant or not, the assumption is made that web pages of a certain topic are most likely linked to other web pages of the same topic [32]. To assess the relevance of a certain web page, a focused crawler often uses techniques from the field of machine learning [16,31]. Classifiers are then leveraged to filter irrelevant content during the crawl process and assign priority on extracted URLs based on the classification result.

#### System Architecture and Processing Workflow

Given the results of the Van der Bosch study [14], it is obvious that sequential processing of such an amount of data would take a tremendous amount of time and/or financial resources. For this reason, a parallel and distributed system architecture as described by Shkapenyuk [33] is necessary to crawl within a reasonable amount of time: to make results available before the web has notably changed. Therefore, such an architecture must be designed to handle thousands of worker threads to fetch web pages in parallel. Besides efficiency in terms of throughput, a crawler should also respect crawler ethics [29,34] (ie, it should honor the robot exclusion protocol [robots.txt]) [29,35,36]; this protocol allows web site administrators to inform the web crawler which parts of a web site should not be processed. In addition, a crawler should not overwhelm the target web server by sending too many request in a short period of time. For this reason, applying a politeness delay (time between requests to the same server) is mandatory. Furthermore, it must be robust protection against so-called spider traps, or web sites containing programmatic errors or dynamically generated links that cause the crawler to be trapped in an infinite loop [29]. Moreover, the HTML parser must tolerate broken and/or invalid markup [29,37,38]. In addition, text extraction components must handle boilerplate detection in an appropriate way [39,40].

**Figure 2:**
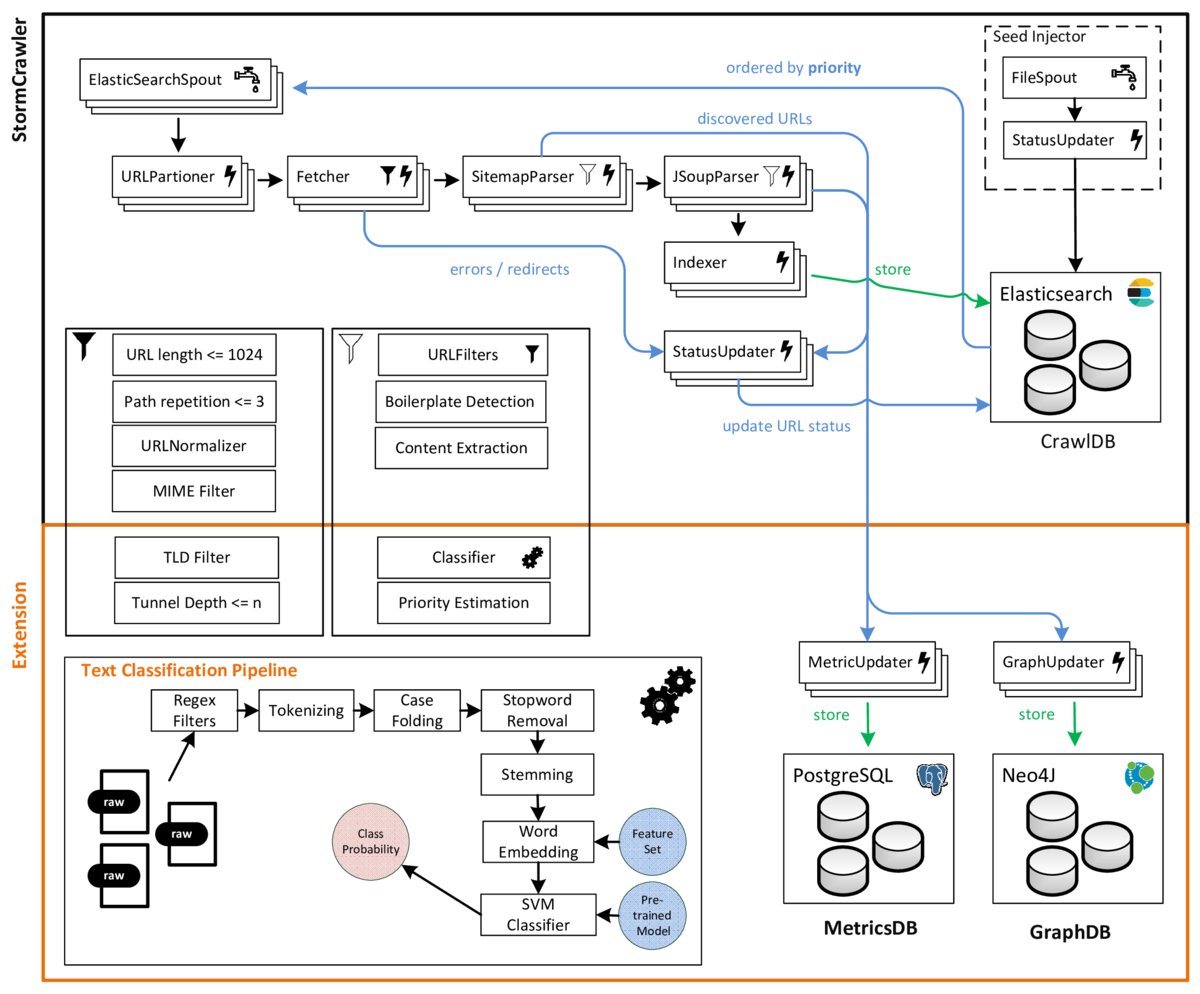
Architecture of a focused crawler based on the StormCrawler software development kit. Spouts (tap symbol) emit data (here: URLs), bolts (lightning symbol) process data (ie fetch, parse, and store the extracted content). Bolts can be enhanced with URL filters (white filter symbol) or parse filters (black filter symbol). URL filters are used to remove URLs based on predefined criteria. Parse filters include URL filters but are primarily used to clean the parsed content and compute topic relevance and priority.

There are several frameworks that realize such distributed crawlers; we built our system on top of the open-source framework StormCrawler [41], a software development kit for building low-latency, scalable crawlers based on the Apache Storm framework [42]. It lacks out-of-the-box components for focused crawling but offers the possibility of adding custom extensions and configuration options. For this reason, we extended it with classifiers and the necessary logic to implement a focused crawler. Figure 2 depicts the architecture of StormCrawler (black) with our focused crawler extension (orange).

The StormCrawler software development kit provides a conventional recursive crawler architecture (upper part of Figure 2); a seed injector is used to read URLs from a text file and adds them to the CrawlDB, which acts as the crawl frontier and content storage. Next, a set of spouts emit yet unseen URLs from the crawl database. To maintain politeness, these URLs are then assigned to cluster nodes (based on their resolved hostname) and directed to the fetchers. The latter will download the respective web pages and forward them to the parsers for link and content extraction; unseen URLs are added to the frontier. Next, the content is sent to the indexers, which store it inside the CrawlDB (in this case an Elasticsearch cluster [43]).

To add focus to StormCrawler, the framework was extended by adding additional bolts and filter components (lower part of Figure 2). After a web page is parsed, the raw text is extracted by using boilerplate detection and XML path language expressions. It is then processed by a text classification pipeline to compute the relevance to the health domain as described by Joachims [44] and Zowalla et al [45]. If a web page is classified as relevant, it is marked for further processing.

Next, a priority value (in this case a value between 0 and 127) is assigned to every URL contained on the given web page [46]. This is done by using (1) the class probability of the current web page [29,32], (2) a check whether the extracted URLs target the same hostname (a web site covering a certain topic will most likely contain more web pages of that topic) [29,32], (3) the anchor text of that link [47], and (4) the link itself using an n-gram approach [48]. Higher priority values will guarantee earlier processing.

In addition, we implemented a soft focused crawling strategy using tunneling to avoid stopping at the first irrelevant page. For example, many front pages of portals may be classified as irrelevant but link to relevant health-related content [28,31]. To do so, a specific filter component tracks the depth and stops after given n steps (eg, n=2, n=3). Irrelevant web pages are not indexed.

To build the web graph of the health domain, during the crawl process a specific bolt keeps track of the visited and discovered links and adds them to a clustered Neo4J graph database. For statistics and metrics related to the crawl, another bolt continuously updates the crawling progress inside a PostgreSQL database. The crawling and classification process is repeated until the frontier is empty or it is stopped manually by the user.

#### System Environment and Hardware Setup

In total, 22 virtual machines participate in the computing cluster providing the infrastructure for the crawler. The corresponding services are used to run, manage, and analyze the crawled web pages on the fly. For this setup, two physical servers of a Cisco unified computing system provide the computational resources and run as a virtualization platform to allow shared resource allocation.

Each server offers two physical central processing units (Intel Xeon E5-2689) with 8 cores each and 256 GB of memory. On the network side, the Cisco unified computing system is attached to two optical 10 gigabit ethernet fibers that provide high bandwidth and ensure scalable throughput. A network attached storage system provides a total disc capacity of 60 TB to persist crawled data and store participating virtual machines via the network file system protocol. This network attached storage is also connected via optical fibers to our university’s core router.

### Evaluation Measures for Focused Web Crawling

Several studies state that the primary metric in evaluating focused crawler performance is the harvest rate [20,29,31,49,50]. Harvest rate is defined as “the fraction of webpages crawled that satisfy the relevance criteria among all crawled webpages” [20]. Previous studies reported that the harvest rate ranges between 10% and 45% for such systems [24,28,31,51].

**Figure 3:**
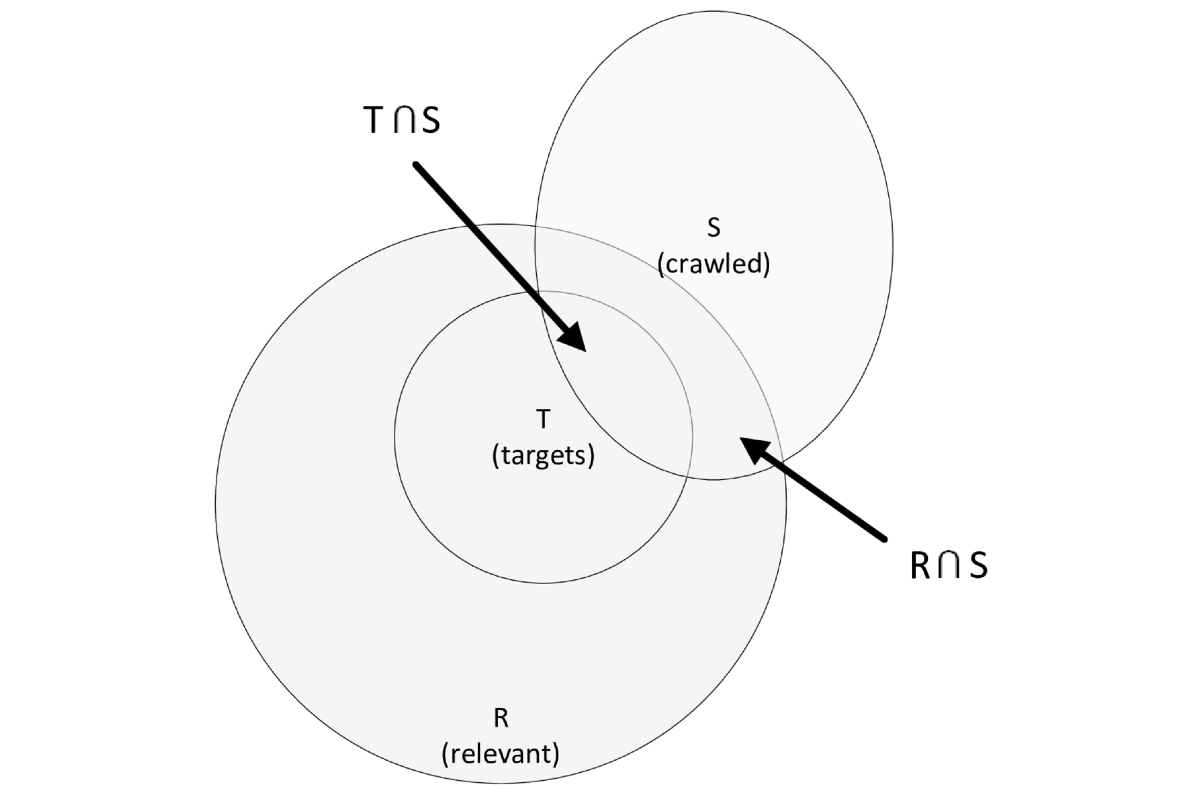
Relationship between target, relevant, and crawled web pages. Recall is estimated based on known relevant target pages and underlying independence assumption.

In addition, the recall (also known as sensitivity) measure can be estimated by using the seed-target approach [29,52,53]. In this context, the initial set of seed pages is split into two sets of which one can be used as seeds and the other as targets (T). Figure 3 depicts the relationship between relevant (R), crawled (S) and target web pages.

**Figure 4:**
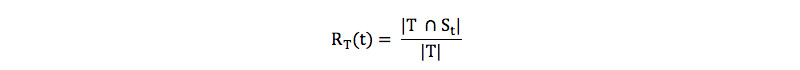
Recall estimate equation.

According to Liu [29], the recall may be estimated if T is a representative, unbiased sample of R independent of the crawling process by the equation in Figure 4 at any time t.

### Text Classification

**Figure 5:**
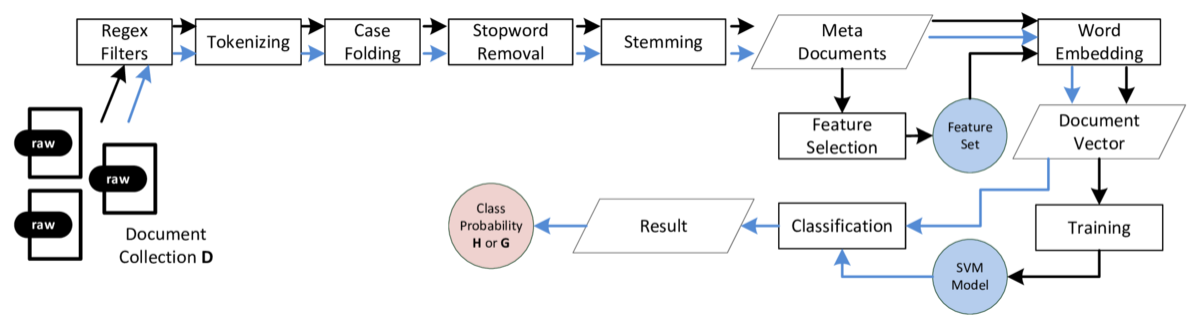
Workflow of an support vector machine–based text classification system: black lines indicate the training process; blue lines indicate the classification process; slanted boxes represent data; rectangular boxes represent computational steps.

Support vector machines (SVMs) originate from the field of machine learning and are known to perform well for text classification tasks [44,45]. For this reason, we relied on an SVM to determine a web page’s relevance within the health domain. Figure 5 depicts the system’s workflow for the training and classification phase.

To build our SVM-based text classifier, we followed related methods as described by Joachims [44]: as a first step toward a health text classifier, automatically gathered health-related articles (contained in a document collection [D]) were cleaned from syntactic markup (eg, boilerplate code, HTML tags). Each article was then tokenized (ie, split into single word fragments) and each character was converted to lower case (also known as case folding). Stop words (eg, the, and, it) were removed as these kinds of tokens do not carry any relevant information. Next, stemming techniques were applied in order to map tokens to their stem forms and reduce morphological variations of words (eg, goes becomes go). Each article was transformed into a document vector containing all distinct terms. To do so, it is necessary to compute the terms that are representative for every article. A so-called feature selection produces a smaller subset of features (F) which yields the most relevant features for each article, limited by a predetermined threshold [54]. Given D and F, an SVM was trained to distinguish between vectors of health-related (H) and non–health-related (G) articles. The resulting classifier may be applied to previously unclassified web pages in order to predict their health-relatedness. To evaluate the classifier’s quality, we used well-established metrics from the field of information retrieval such as accuracy, recall, and precision [29].

LIBSVM [55] and its object-oriented binding zlibsvm [56] were used as an SVM implementation of the text classifier. For building and training the SVM, the process described by Joachims [44] was applied. To reduce dimensionality, the feature selection method *information gain* was used [54]. Word embedding was conducted using *tfc* [57] as a term-weighting approach.

To find an optimal hyperparameter combination for the chosen radial basis function kernel, a grid search using 10-fold cross-validation, as recommended by the LIBSVM authors [55], was conducted. According to the Pareto Principle, training and test data were constructed using an 80:20 split [58]. In addition, the classes inside these data sets were equally balanced according to Wei and Dunbrack [59] as the real-world class distribution of H and G is unknown.

### Graph Metrics

The graph structure of the web has been extensively analyzed in several studies [15,17,60,61]. In this context, a graph node represents a web page and an edge represents a link between two web pages. We generated a so-called host-aggregated graph from the original web graph in order to reduce its computational complexity and explore its properties [61]. In this process, single web pages are combined and represented by their parent web site (including outgoing and ingoing links). On the resulting host-aggregated graph, we applied the following metrics:

- Average degree is the average number of edges connected to a node [62]. For a (directed) web graph, this is defined as the total number of edges divided by the total number of nodes. The average in-degree denotes the average number of ingoing edges to a node (ie, links to a web site). The average out-degree is defined as the average number of outgoing edges of a node (ie, links targeting other web sites).
- Modularity measures the strength of division of a graph into clusters or groups [62,63]. Graphs with a high modularity have dense connections between the web sites within certain clusters but sparse connection to other web sites, which are contained in different clusters.
- PageRank is a centrality-based metric that allows identification of web sites (nodes) of importance inside a graph [64]. The underlying assumption is that an important or prestigious web site will receive more links from other important web sites (ie, higher in-degree).

Other metrics such as network diameter and the average path length (ie, the average number of clicks which will lead from one web site to another) are frequently used for graph analysis [62,65].

### Data Acquisition

#### Seed Generation

The selection of seed sources is crucial for the performance of a focused crawler [24,28,66-68]. For certain top-level domains (TLDs; eg, .com), the domain name system zone files are available to the public free of charge containing all registered domains for the given TLD. These zone files can then be used to extract seeds. However, due to data protection regulations, accessing and using the domain name system zone files for the ccTLDs .de, .at, and .ch was not possible.

Other studies leverage search engines with specific queries [28,66,69] to obtain high-quality seeds. However, most search engines restrict the amount of queries and limit the returned amount of results. Also, the results might be influenced by commercial interests and crafting high-quality search queries demands time and/or financial resources.

Another widely used seed source is the web taxonomy Curlie [22,29,31,66,70,71], which provides human-maintained precategorized web sites. Seeds can be harvested as dumps and are available free of charge. In addition, it is possible to reuse the results of a previous crawl to generate seeds. For this study, we relied on Curlie and the data of a previous health-related crawl conducted in 2016 [72].

#### Machine Learning Data Sets

##### Training and Test Corpus

To obtain a large enough data set for training and testing the SVM text classifier used within the focused crawler, web pages from various German content providers were obtained. First, the web pages were downloaded by specialized web crawlers implemented in Java using the crawler4j framework [73]. Next, boilerplate detection and data cleaning were conducted using regular expression filters. After this step, the cleaned textual content was stored in a relational database for further processing. Regarding each content provider, a random sample was manually inspected by the authors in order to assess data quality.

Each content provider and all related articles were put into one of the two classes: health-related language (H) or general language (G). The coding was based on (1) the organizations providing the content, (2) health-related content certification (eg, Health On the Net Foundation Code of Conduct), and (3) a manual inspection by the authors, in which the topic relevance of a random sample for each content provider was assessed.

##### Crowd-Validated Test Corpus

As the training and test corpus were generated by using a priori knowledge of each content provider, the authors decided to construct an additional independent human-validated data set to evaluate the classifier’s performance.

**Figure 6:**
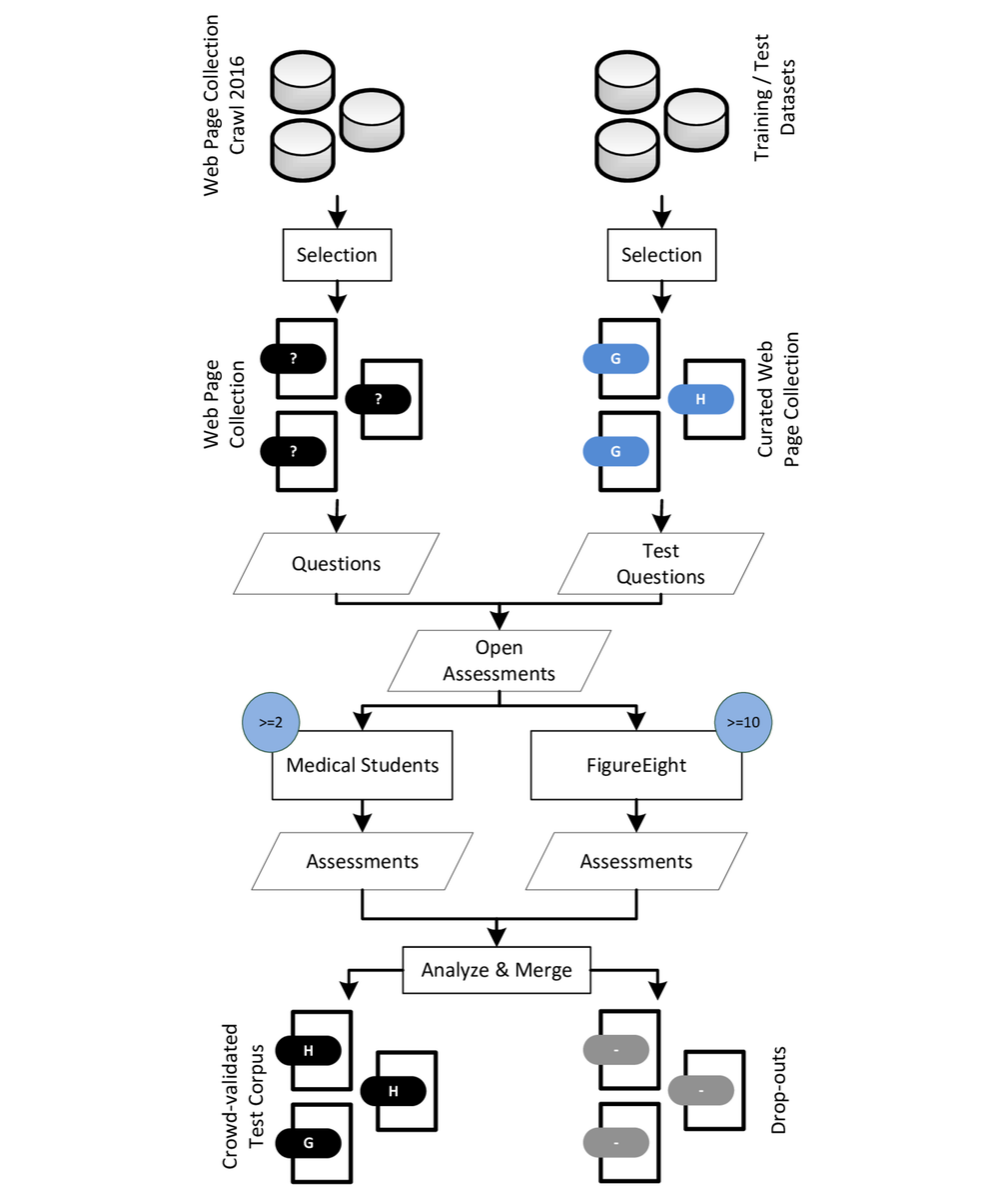
Workflow of the crowd-sourcing approach to build a test corpus for the purpose of classifier evaluation. Black lines indicate the assessment process; slanted boxes represent data; rectangular boxes represent processing steps.

Recent studies have shown that crowdsourcing can produce comparable results to human experts at a faster pace [74-78]. Thus, crowdsourcing was used to assess the evaluation data set. Figure 6 depicts the process of building this validated data set.

First, web pages were manually selected from a crawl conducted in 2016 [72]. It was ensured that the selected pages were neither included in the training set nor in the test corpus generated in the previous step. Next, each web page was assessed by a group of workers and categorized as H or G. Raters were given clear instructions on how to categorize given web pages (see Multimedia Appendix 1). In addition, each rater successfully completed a quiz-based training before they could participate in the study [79-83]. Precategorized web pages (test questions) were mixed into the rating process as test questions to keep the attention of the raters at a high level.

If a rater failed to answer a specific amount of such test questions, the assessments of this rater were considered as dropouts. Following the recommendation by Carvalho et al [84], each web page was assessed by at least 10 crowd-workers on the commercial crowd-working platform FigureEight [85]. In addition, the same web pages were coded by final year medical students (at least two students per web page) at the University of Heidelberg in the context of the lecture Medical Informatics. Study participation was voluntary.

If there was no clear majority vote for a certain class between the crowd-workers, the assessments of the medical students were taken into consideration. If there was still no agreement, the web page was listed as a dropout.

The statistical software R version 3.4.4 (R Foundation for Statistical Computing) on an Ubuntu 18.04 LTS 64-bit computer was used to compute percent agreement [86] and Fleiss κ [87].

## Results

### Seeds

Seeds were obtained from the health category (German: Gesundheit) of Curlie and a health-related crawl conducted in 2016 targeting health-related web sites in German [72]. In total, n=22,405 seeds with ccTLDs .de (19,126/22,405, 85.36%), .at (1530/22,405, 6.83%), .ch (1749/22,405, 7.81%) were collected and used in this study. The full list of seeds can be found in Multimedia Appendix 2.

### Machine Learning Data Sets

#### Data Set Characteristics

Web pages from various German content providers were collected between April 24, 2018, and August 16, 2018. A detailed list and description of each content provider is shown in Table 1. In total, 98,442 articles were collected. The average word count for each document was 741; the average sentence count was 44.

For category H, we collected 9638 articles from the categories “medicine” and “medical report” from Deutsches Ärzteblatt (a magazine tailored to physicians) and 1907 from Apotheken Umschau (a magazine freely available in German pharmacies, tailored to lay citizens). In addition, we acquired 235 and 636 articles from the medical content providers Institute for Quality and Efficiency in Healthcare and Onmeda, respectively. Moreover, 2829 documents were obtained from the national health portal of the Republic of Austria. In addition, 28,436 health-related articles from Wikipedia Health were gathered by using the Wikipedia category graph. For category G, 18,364 random articles from Wikipedia General were collected, which were not related to the category Health (German: Gesundheit). In addition, 36,297 German web pages were selected randomly from the Common Crawl Foundation.

**Table 1:** Total number of acquired articles and respective class labels of various German content providers.

Content provider	Class	Cert^a^	Organization	Articles	Words (mean)	Words (median)	Sentences (mean)
Wikipedia Health	H^b^	no	Wikimedia Foundation	28,436	429	254	31
Wikipedia General	G^c^	no	Wikimedia Foundation	18,364	736	266	26
Common Crawl	G	no	Common Crawl Foundation	36,297	480	429	33
Deutsches Ärzteblatt	H	no	German Medical Association, National Association of Statutory Health Insurance Physicians	9638	1852	520	136
Onmeda	H	yes	Gofeminin.de GmbH	636	6564	6113	439
gesundheitsinformation.de	H	yes	Institute for Quality and Efficiency in Healthcare	235	1923	1799	139
Apotheken Umschau	H	yes	Wort & Bild Verlag	1907	1052	658	73
GESUNDheit.gv.at	H	no	Ministry of Social Affairs (Austria)	2929	295	221	21
Total	—^d^	—	—	98,442	741	339	44

^a^Yes indicates that a provider is certified by the Health On The Net Foundation Code of Conduct or another certification provider.

^b^H: health-related language.

^c^G: general language.

^d^Not applicable.

#### Training and Test Corpus

For training and evaluation of the SVM classifier, 87,562 articles were used. Table 2 lists the final data sets. In total, 80.00% (70,048/87,562) of articles were used for training the classifier and 20.00% (17,514 of 87,562) were used for testing.

**Table 2:** Total amount of articles used in the training and test corpus per content provider with corresponding class labels: health-related language (H) and general language (G).

Content provider	Class	Documents		
		Training	Test	Total
Wikipedia	H^a^	22,748	5688	28,436
Wikipedia	G^b^	10,339	2585	12,924
Common Crawl	G	24,685	6172	30,857
Deutsches Ärzteblatt	H	7710	1928	9638
Onmeda	H	509	127	636
gesundheitsinformation.de	H	189	46	235
Apotheken Umschau	H	1525	382	1907
GESUNDheit.gv.at	H	2343	586	2929
Total	–^c^	70,048	17,514	87,562

^a^H: health-related language.

^b^G: general language.

^c^Not applicable.

#### Crowd-Validated Test Corpus

A total of 432 web pages (216 per class) were manually selected from a health-related crawl conducted in 2016 [72]. The selected web pages were neither contained in the training nor in the test corpus (see Table 2).

Each web page was assessed by 10 crowd-workers between February 2, 2019, and February 16, 2019, on the commercial crowd-working platform, FigureEight [85]. In total, 4367 assessments by 28 crowd-workers were collected at a cost of US $36.06. The overall satisfaction (as measured by FigureEight) was 4.45 out of 5 possible points (instructions clear: 4.5/5; test questions fair: 4.55/5; ease of job: 4.5/5; payment: 3.65/5); 14 out of 28 (50%) workers participated in this voluntary exit survey. Percent agreement was 0.855; Fleiss κ was 0.279.

In addition, the same web pages were coded by medical students (n=40). Study participation was voluntary. Each web page was assessed by at least two students. Percent agreement was 0.719; Fleiss κ was 0.337. According to Landis and Koch [88], these κ values correspond to a fair agreement.

The resulting data set contained n=384 web pages (192 per class). This corresponds to a dropout rate of 11.1% (48/432). The full list of coded web pages is given in Multimedia Appendix 3.

### Classifier Performance

The classifier was evaluated against the test and crowd-validated data set, and the results are presented in Table 3. The classifier achieved a precision of 0.934, a recall of 0.940, and an accuracy of 0.937 on its test data set; 5.96% (522/8757) of health-related web pages were falsely classified as nonrelevant by the SVM. On the other hand, 6.57% (575/8757) of the nonrelevant pages were classified as health-related.

On the crowd-validated real-world data set, the classifier achieved an accuracy of 0.966, a precision of 0.954, and a recall of 0.989. Only 1.0% (2/192) of the health-related web pages were falsely classified as nonrelevant, and 5.7% (11/192) of nonrelevant web pages were classified as health-related.

**Table 3:** Listing of the confusion matrix and related evaluation metrics for the test and crowd-validated data set.

Evaluation data sets		Baseline					
		Health	General	Sum	Accuracy	Precision	Recall
**Test data set**					0.937	0.934	0.94
	SVM^a^	—^b^	—	—	—	—	—
	Health	8182	575	8757	—	—	—
	General	522	8235	8757	—	—	—
	Sum	8704	8810	17,514	—	—	—
**Crowd-validated data set**					0.966	0.954	0.989
	SVM	—	—	—	—	—	—
	Health	181	11	192	—	—	—
	General	2	190	192	—	—	—
	Sum	183	211	384	—	—	—

^a^SVM: support vector machine.

^b^Not applicable.

### Crawler Performance

Our system achieved a download rate of 7 to 10 documents per second. This sums up to 227 days of pure crawling and classification of approximately 133 million web pages.

**Figure 7:**
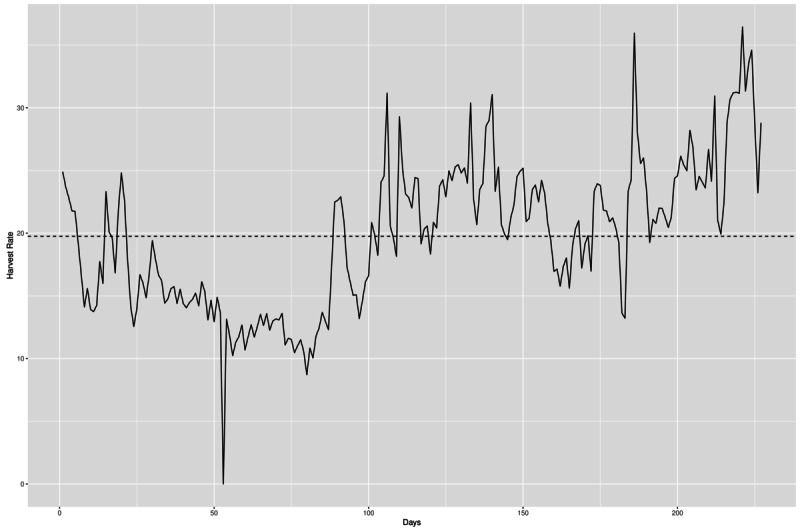
Harvest rate over time measured at the end of each day (dashed line represents the mean harvest rate). Note that the drop at day 53 is related to an outage at our data center. Peak at day 106: storm cluster was extended by three additional virtual machines. Peaks at days 157, 158, 191, 194 and 222: crawl was resumed after infrastructure maintenance due to urgent security updates that required a restart of the host system and/or of the virtual machines.

The crawl yielded approximately 13.5 million presumably relevant web pages and approximately 119.5 million nonrelevant web pages. Figure 7 depicts the harvest rate during the crawl.

The overall mean harvest rate was 19.76% (HR_*t* =222_=HR_max_=36.45%; HR_*t* =53_=HR_min_=0.00%). HR_max_ was achieved at day 222 as the crawl was resumed after infrastructure maintenance due to urgent security updates; HR_min_ was recorded on day 53. It was caused by a data center outage in which the infrastructure had to be shut down.

As an additional measure, we estimated the recall of our focused crawling by using the seed-target approach [29]. For this purpose, the initial seed set (n=22,405) was divided into a set of seeds (n=17,405) and targets (n=5000); ccTLD distribution was maintained in each sub set, and 4105 out of 5000 targets (82.10%) were contained in the crawl. This corresponds to an estimated recall of 0.821.

### Graph Structure

The graph database Neo4J in v3.5.4 and its graph algorithm plugins [89] were used to compute the metrics as described in Graph Metrics on an Ubuntu 18.04 LTS 64-bit server. In order to reduce graph complexity, all web pages belonging to the same web site were aggregated and substituted by their parent web site (including outgoing and ingoing links; see Graph Metrics). The resulting graph contains 215,372 nodes (web sites) and 403,175 edges (links between web sites). A total of 82.56% (177,816/215,372) of the web sites belong to the ccTLD .de; 7.95% (17,126/215,372) to .at, and 9.49% (20,430/215,372) to .ch.

The graph has a network diameter of 25. The average path length is 6.466. The average degree is 1.872, the average in-degree is 1.892, and the average out-degree is 1.845. Modularity was computed to be 0.723.

During the analysis, several types of website publishers emerged: public institutions, nonprofit organizations, and private organizations or single individuals. As the ccTLD .de has the highest share within the graph, a global ranking according to PageRank would be dominated by .de web sites. For this reason, the following paragraph will present the top 25 web sites according to PageRank for each ccTLD separately.

Table 4 lists the 25 top-ranked web sites according to PageRank with their respective publisher for .de; 12 out of 25 (48%) are published by public institutions, 32% (8/25) are published by nonprofit organizations, and 20% (5/25) by private organizations. The top-ranked 25 web sites for .at are shown in Table 5; 12 out of 25 (48%) are published by public institutions, 4% (1/25) are published by nonprofit organizations, and 48% (12/25) by private organizations (see Table 5). For the ccTLD .ch, 24% (6/25) are published by public institutions, 40% (10/25) originate from nonprofit organizations, and 9/25 (36%) are published by private organizations (see Table 6).

Overall, 40% (30/75) are web sites published by public institutions, 25% (19/75) are published by nonprofit organizations, and 35% (26/75) by private organizations.

**Figure 8:**
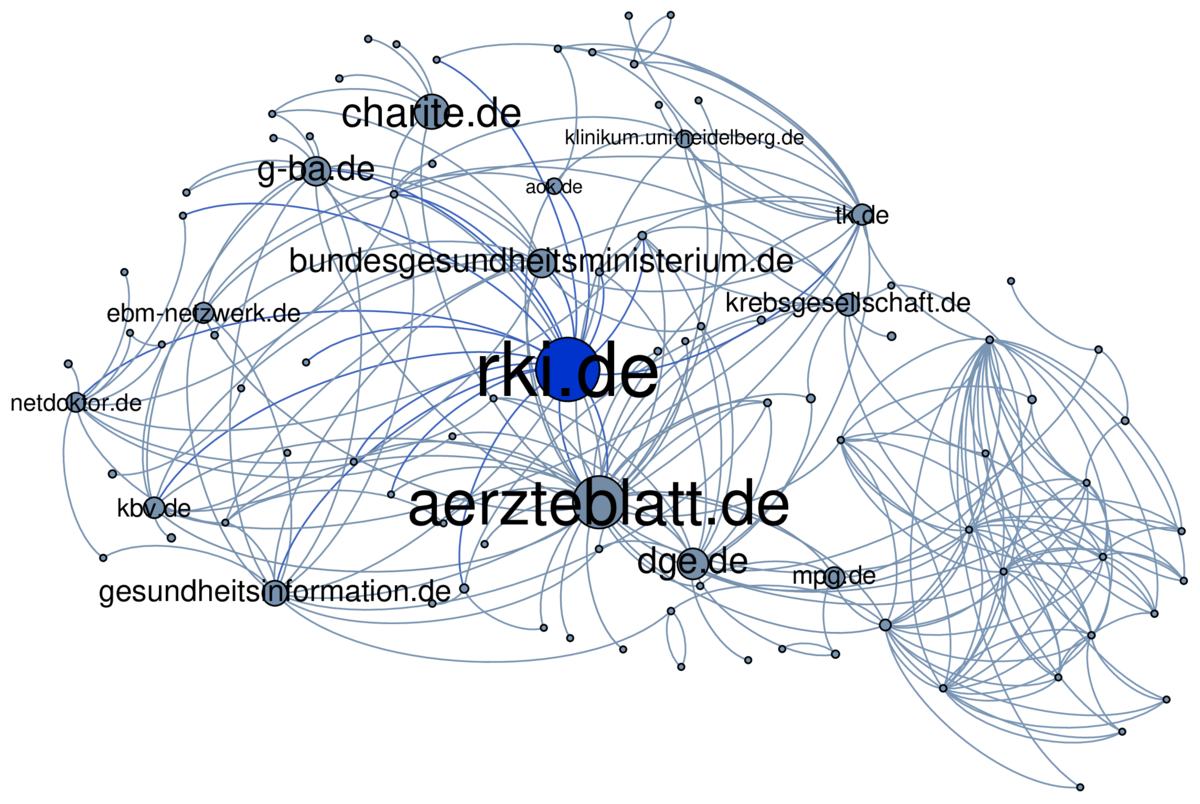
A small extract of the host-aggregated web graph with focus on the website www.rki.de. The surrounding nodes represent websites with a maximum link-distance of two starting from www.rki.de. An edge between two nodes implies that there exists at least one hyperlink between some web pages of the hosting websites in either way. Only those websites are included whose content is highly health-related (ie, which were automatically classified as belonging to H with a probability equal to or greater than 0.93). Moreover, they have at least one ingoing and one outgoing link. The bigger a node and its caption, the higher is its page rank. For illustration reasons, directional arrows were not included.

The graph visualization tool Gephi v0.9.2 [90] was used on a bare-metal Windows 10 64-bit computer to explore the host-aggregated graph structure. Unfortunately, we experienced serious performance issues while running Gephi’s visualization algorithms. This is a main reason why we illustrate just a small example extract of the host-aggregated graph: Figure 8 consists of 94 nodes and 243 edges and presents basic aspects of the graph’s structure. The focus is on www.rki.de as the top-ranked web site for the ccTLD .de (according to our analysis from below). The surrounding nodes represent health-related web sites in close proximity of www.rki.de.

**Table 4:** Domains of 25 top-ranked web sites for country-code top-level domain .de with their respective publisher according to PageRank.

Rank	Domain	Publisher	Type
1	www.rki.de	Robert Koch Institute	PI^a^
2	www.aerzteblatt.de	Deutscher Ärzte-Verlag GmbH	PI
3	www.charite.de	Charité–Berlin University of Medicine	PI
4	www.deutsche-alzheimer.de	Deutsche Alzheimer Gesellschaft	NPO^b^
5	www.aerztezeitung.de	Springer Medizin Verlag GmbH	PO^c^
6	www.dge.de	Deutsche Gesellschaft für Ernährung	NPO
7	www.g-ba.de	Gemeinsamer Bundesausschuss (Federal Joint Comitee)	PI
8	www.bzga.de	Bundeszentrale für gesundheitliche Aufklärung (Federal Centre for Health Education)	PI
9	www.bundesgesundheitsministerium.de	Bundesministerium für Gesundheit (Federal Ministry of Health)	PI
10	www.apotheken-umschau.de	Wort & Bild Verlag	PO
11	www.dimdi.de	Deutsches Institut für Medizinische Dokumentation und Information (German Institute for Medical Documentation and Information)	PI
12	www.gesundheitsinformation.de	Institut für Qualität und Wirtschaftlichkeit im Gesundheitswesen (Institute for Quality and Efficiency in Healthcare)	PI
13	www.osteopathie.de	Verband der Osteopathen Deutschland eV	NPO
14	www.krebsgesellschaft.de	Deutsche Krebsgesellschaft eV	NPO
15	www.bfarm.de	Bundesinstitut für Arzneimittel und Medizinprodukte (Federal Institute for Drugs and Medical Devices)	PI
16	www.kbv.de	Kassenärztliche Bundesvereinigung	PI
17	www.krebshilfe.de	Stiftung Deutsche Krebshilfe	NPO
18	www.tk.de	Techniker Krankenkasse (Health Insurance)	PO
19	www.ebm-netzwerk.de	Deutsches Netzwerk Evidenzbasierte Medizin eV	NPO
20	www.bmg.bund.de	Bundesministerium für Gesundheit (Federal Ministry of Health)	PI
21	www.netdoktor.de	NetDoktor.de GmbH	PO
22	www.drk.de	Deutsches Rotes Kreuz eV (German Red Cross)	NPO
23	www.herzstiftung.de	Deutsche Herzstiftung	NPO
24	www.klinikum.uni-heidelberg.de	Universitätsklinikum Heidelberg	PI
25	www.aok.de	AOK Gesundheiskasse (Health Insurance)	PO

^a^PI: public institution.

^b^NPO: nonprofit organization.

^c^PO: private organization.

**Table 5:** Domains of 25 top-ranked web sites for country-code top-level domain .at with their respective publisher according to PageRank.

Rank	Domain	Publisher	Type
1	www.gesundheit.gv.at	Bundesministerium für Arbeit, Soziales, Gesundheit und Konsumentenschutz (Ministry of Social Affairs)	PI^a^
2	www.meduniwien.ac.at	University of Vienna	PI
3	www.bmgf.gv.at	Bundesministerium für Arbeit, Soziales, Gesundheit und Konsumentenschutz (Ministry of Social Affairs)	PI
4	www.sozialministerium.at	Bundesministerium für Arbeit, Soziales, Gesundheit und Konsumentenschutz (Ministry of Social Affairs)	PI
5	www.apotheker.or.at	Österreichische Apothekenkammer (Austrian Pharmaceutical Association)	PI
6	www.sam-pharma.at	Pharma Handel GmbH	PO^b^
7	www.aerztekammer.at	Österreichische Ärztekammer (Austrian Medical Association)	PI
8	www.univie.ac.at	University of Vienna	PI
9	www.herz-ambulatorium.at	Individual Person	PO
10	www.herz-ordination.at	Individual Person	PO
11	www.tg-steiermark.at	TG Therapeutische Gemeinschaft Betriebs GmbH	NPO^c^
12	www.impuls-fs.at	Institut für medizinisch-physiotherapeutische Untersuchung, Lehre und Schulung	PO
13	www.medunigraz.at	University of Graz	PI
14	www.brustvergroesserung-leicht.at	Individual Person	PO
15	www.bmg.gv.at	Bundesministerium für Arbeit, Soziales, Gesundheit und Konsumentenschutz (Ministry of Social Affairs)	PI
16	www.kages.at	Steiermärkische Krankenanstaltengesellschaft mbH	PO
17	science.orf.at	Österreichischer Rundfunk (Austrian Broadcasting Corporation)	PI
18	www.gynmed.at	Individual Person	PO
19	www.fhstp.ac.at	St. Pölten University of Applied Sciences	PI
20	www.dr-boehm.at	Individual Person	PO
21	bmg.gv.at	Bundesministerium für Arbeit, Soziales, Gesundheit und Konsumentenschutz (Ministry of Social Affairs)	PI
22	www.novartis.at	Novartis AG	PO
23	www.babyforum.at	FOKUS KIND Medien, CRAFT & VALUE	PO
24	femmestyle.at	Schönheitschirurgie femmestyle	PO
25	www.pfizer.at	Pfizer Inc	PO

^a^PI: public institution.

^b^PO: private organization.

^c^NPO: nonprofit organization.

**Table 6:** Domains of 25 top-ranked web sites for country-code top-level domain .ch with their respective publisher according to PageRank.

Rank	Domain	Publisher	Type
1	www.uzh.ch	University of Zurich	PI^a^
2	www.usz.ch	Universitätsspital Zürich	PI
3	www.srf.ch	Schweizerische Radio- und Fernsehgesellschaft (Swiss Broadcasting Corporation)	PI
4	www.netdoktor.ch	netdoktor GmbH	PO^b^
5	www.pancreas-help.ch	Schweizer Selbsthilfeorganisation Pankreaserkrankungen	NPO^c^
6	www.mutterglueck.ch	Individual Person	PO
7	www.association-osteo-swiss.ch	Schweizerischer Verband der Osteopathen	NPO
8	www.unibas.ch	University of Basel	PI
9	www.ethz.ch	ETH Zurich (Swiss Federal Institute of Technology in Zurich)	PI
10	www.rheumaliga.ch	Rheumaliga Schweiz	NPO
11	www.lungenliga.ch	Lungenliga Schweiz	NPO
12	www.rotpunkt-apotheken.ch	Rotpunkt-Pharma AG	PO
13	www.pharmawiki.ch	PharmaWiki GmbH	PO
14	www.bayer.ch	Bayer AG	PO
15	www.patientensicherheit.ch	Stiftung Patientensicherheit Schweiz	NPO
16	saez.ch	EMH Schweizerischer Ärzteverlag AG	NPO
17	www.swissheart.ch	Schweizerische Herzstiftung	NPO
18	gesundheitsfoerderung.ch	Gesundheitsförderung Schweiz	NPO
19	sensomotorische-lebensweisen.ch	Individual Person	PO
20	www.spitaluster.ch	Spital User	PO
21	symptome.ch	NOXA GmbH	PO
22	www.meineimpfungen.ch	Stiftung meineimpfungen	NPO
23	unicef.ch	United Nations International Children's Emergency Fund	NPO
24	www.bauchtumor.ch	Universitätsspital Bern	PI
25	www.fettabsaugungen.ch	FSnD Ltd	PO

^a^PI: public institution.

^b^PO: private organization.

^c^NPO: nonprofit organization.

## Discussion

### Principal Findings

One aim of the study was to demonstrate the suitability of a focused crawler approach for the acquisition of health-related content. Our system achieved an average harvest rate of 19.76% during the entire crawl. In addition, the results show that the majority of the target seeds (4105/5000) could be obtained, which corresponds to a recall of 0.821. Therefore, we are confident that the proposed method is suitable to acquire most health-related content on the web and generate a suitable domain-specific graph representation.

A first manual investigation of several hundred randomly selected pages suggests that our approach produces accurate results. The results indicate that the web sites and web pages of major German, Austrian, and Swiss health-related public institutions have indeed been discovered, even though they were not contained in the initial seeds.

With respect to the study aims 2 and 3, we were able to provide a curated list of 22,405 seed points for the health domain in the D-A-CH region extracted from Curlie (see Multimedia Appendix 2). In addition, a data set with 396 items was created and evaluated by crowd-workers that can be used by other researchers to evaluate similar text classifiers (see Multimedia Appendix 3).

A first analysis of the graph structure (see study aim 4) shows that public institutions and nonprofit organizations have a higher importance according to their PageRank than web sites of private players inside the GHW.

### Limitations

Several limitations apply for this study. First, we are not sure whether the seed pages cover a broad spectrum of topics within the health domain as we only acquired seeds from Curlie and a previous health-related crawl [72]. Using specifically crafted queries against established search engines would have increased the amount of available seeds and could have influenced the crawl in a positive way [28,66,69]. However, due to limited amounts of resources and time, we did not follow this approach as the web taxonomy Curlie and a previous crawl gave faster access to seed URLs. As Curlie is a community-driven web taxonomy, the publication process of new URLs is not strictly regulated. This might be a reason for the high share of private players within the top ranks of the web graph as everybody is eligible to publish a web site’s URL on Curlie. In addition, the community behind Curlie is rather small compared with its predecessors (ie, URLs pointing to rather new content providers might not be contained in it). Therefore, corresponding web pages and their out-links might have been missed during the crawling process. This implies that reported graph properties might have been influenced by the chosen seed sources.

Second, with a mean accuracy of 0.951, our classifier might have produced false positive results during the crawl process. Third, we only considered the ccTLDs .de, .at, and .ch to avoid the need for a language classification system, as most web sites on these ccTLDs are written in German. Therefore, the data crawled covers only a certain fraction of the GHW, for example, as web sites in German published under .org are not contained.

### Comparison With Prior Work

Previous studies investigated the use of focused crawler techniques to harvest biomedical or health-related text material [27,28]. In both analyses, the authors report that the use of focused crawlers requires a lot of computational effort to collect the data and analyze it in an appropriate way, which we can confirm by our observations.

Compared with the study by Rheinländer et al [28] in which they report an harvest rate of 38%, our system achieved an harvest rate of only 19.76%. This might be caused by (1) our system using a soft-focused crawling strategy meant it did not stop at the first encountered irrelevant web page, leading to an increase in irrelevant web pages and crawling time and (2) our crawl was limited to the ccTLDs .de, .at, and .ch as we did not implement a language classifier. This might have influenced the harvest rate of our system as well, yet it achieved a harvest rate in the typical range for such systems [24,28,31,51] (see Related Work).

In contrast to the studies by Rheinländer et al [28] and Amalia et al [27], we focused on the German language and the GHW. This study contributes to the field by demonstrating the suitability of a focused crawler approach for the acquisition of German health-related content in the D-A-CH region. A secondary study outcome is a curated list of seed points for the health domain in the D-A-CH region (see Multimedia Appendix 2). In addition, the crowd-validated evaluation data set (see Multimedia Appendix 3) can be used to evaluate other text classifiers for the given purpose. Moreover, this study gives first insights regarding the graph structure of the health-related web in the D-A-CH region.

### Conclusions and Further Research

In this study, a system was presented which uses a focused crawling approach to gather the structure of the GHW. The system used an SVM-based classifier that was trained to assess the relevance of a web page for the health domain. The results indicate that the presented focused crawler is a suitable method for acquiring large health-related textual datasets and can be used to generate domain-specific graph representations. In future work, the authors intend to expand their web crawl by leveraging seed lists generated via search engine providers.

We also plan to analyze the linguistic characteristics of the crawled data as well as identify important topics and trends within this data. This will also include the identification of credible content providers and a comparison of the health-related web between Germany, Austria, and Switzerland. Moreover, future work will include a deeper exploration and analysis as well as a visualization of the resulting graph structure. Using these insights and with the acquired data available, an implementation and evaluation of a health-specific search engine for information seeking citizens will be possible.

## Appendix

Multimedia Appendix 1 Instruction for raters written in German.

Multimedia Appendix 2 List of seed points.

Multimedia Appendix 3 Crowd-annotated corpus of health-related and non–health-related web pages.

## Abbreviations

ccTLD: country-code top-level domain
D-A-CH: Germany, Austria, and Switzerland
GHW: German Health Web
SVM: support vector machine
TLD: top-level domain
