# Supplementary material for: Crawling the German Health Web: Exploratory Study and Graph Analysis
Source: J Med Internet Res. 2020 Jul 24;22(7):e17853. doi: 10.2196/17853 (PMC7414401; doi:10.2196/17853)
Supplement: Multimedia Appendix 1 [file jmir_v22i7e17853_app1.docx]

Multimedia Appendix 1:

Instruction for raters in German language.

Einführung

Im Rahmen dieser Untersuchung sollen Webseiten bzw. deren Inhalt in die Kategorien „Enthält Gesundheitsinformationen“ und „Enthält keine Gesundheitsinformationen“ eingeteilt werden.

Eine **Definition,** welche Inhalte im Rahmen dieser Untersuchung als Gesundheitsinformation betrachtet werden sollen, finden Sie im Abschnitt „Definition“. Entsprechende Beispiele aus beiden Kategorien finden Sie im Abschnitt „Beispiele“.

Aufgabenbeschreibung

*Ihre Aufgabe besteht* darin, den **angezeigten Text** entweder in die Kategorie „Enthält *Gesundheitsinformationen“* oder *„Enthält keine Gesundheitsinformationen“* einzuordnen, nachdem Sie den entsprechenden Text vollständig gelesen haben.

Zu diesem Zweck werden Ihnen jeweils die Quelle sowie ein dazugehöriger Text angezeigt. Beurteilen Sie bitte **ausschließlich** den dargestellten Text. Besuchen Sie bitte **keine** im Text verlinkten Webseiten, auch **nicht** die Quelle selbst.

**Weiterführende Bearbeitungshinweise**

Die zu beurteilenden Texte wurden mittels maschineller Verfahren erfasst und automatisiert von Auszeichnungssprachen (z.B. HTML) bereinigt. Wir können jedoch nicht vollständig ausschließen, dass die Texte ggf. Störartefakte enthalten oder schlecht formatiert sind. Ebenfalls können orthografische oder stilistische Mängel in den Texten vorhanden sein.

Versuchen Sie in einem solchen Fall dennoch eine für Sie stimmige Entscheidung zu treffen.

**Definition von "Gesundheitsinformationen"**

Im Internet existieren Informationen zu Gesundheits- und Krankheitsfragen in vielfältigen Formen, z.B. als Broschüren zur gesunden Ernährung, Ratgeber zu Früherkennungs-untersuchungen oder Aufklärungsschriften zu medizinischen Behandlungen. Es finden sich aber auch Informationen zu Symptomen, Krankheiten sowie deren Behandlungsoptionen. Aber auch Laien, wie beispielsweise Patienten und Angehörige, erzeugen Inhalte und tauschen sich zum Themengebiet „Gesundheit“ in Social Media, Internetforen oder Selbsthilfegruppen aus.

Aus diesem Grund ist der Begriff „Gesundheitsinformation“ im Rahmen dieser Untersuchung sehr offen definiert und umfasst u.a. die folgenden Themenkomplexe:

- Krankheiten
- Diagnosen, Therapien oder Behandlungen
- Pharmazeutische Informationen (z.B. zu Medikamenten, ...)
- Homöopathie
- Ernährung & Sport & Lifestyle Informationen für ein "gesundes" Leben (Prävention)
- Informationen zu Krankenhäusern, Arztpraxen, etc.
- Informationen von und über Selbsthilfegruppen
- Von Patienten oder Nutzern erzeugter Inhalt zum Thema Gesundheit, z.B. in Social Media oder Internetforen

**Ergänzende Hinweise:**

Informationen zum Gesundheitszustand von Tieren oder deren Behandlung (Veterinärmedizin) sind im Rahmen dieser Untersuchung als keine Gesundheitsinformationen zu betrachten.

**Text-Beispiele zu "Gesundheitsinformationen"**

Die nachfolgenden Textausschnitte enthalten „Gesundheitsinformationen“:

*URL: https://de.wikipedia.org/wiki/Nasensekret*

*Der biologische Nutzen des Nasensekrets besteht in der Befeuchtung der Atemluft und ihrer Reinigung von Staub. Der Hauptteil des Nasensekrets ist dünnflüssig und fließt fortwährend nach innen über den Rachenraum ab. Bei Menschen und anderen Primaten, die zur Unterordnung der Trockennasenaffen gehören, verfestigt sich jedoch im Bereich der Nasenlöcher, der verstärkt der Austrocknung ausgesetzt ist, ein Teil des Sekrets und kann nicht mehr von alleine abfließen. Dieses angetrocknete Sekret muss dann, z. B. mit Hilfe eines Fingers („Nasebohren“, „Popeln“) oder durch Ausblasen der Nase in ein Taschentuch („Schnäuzen“), nach außen entfernt werden, um wieder eine freie Atmung durch die Nase zu ermöglichen.*

***URL: https://www.klinikum.uni-heidelberg.de/Radiologie.118379.0.html***

*Die klinische Versorgung umfasst die Durchführung und Befundung der diagnostischen und interventionellen radiologischen Untersuchungen (Projektionsradiographie, Durchleuchtung, Computertomographie, Magnetresonanztomographie, Knochendichtemessung), die Beteiligung an der Versorgung mittels Ultraschall sowie die Durchführung der klinisch-radiologischen Konferenzen.*

***URL: https://www.apotheken-umschau.de/ruecken/schmerzen-im-gesaess-kreuz***

*Ärzte nennen ständiges Sitzen "statische Überbelastung". In der Tat kommen dabei erhebliche Gewichte zum Tragen. Im Sitzen lastet der gesamte Oberkörper nebst Kopf und Armen auf der Lendenwirbelsäule und dem Becken – anderthalb mal so viel wie im Stehen. Dass Gesäßschmerzen (Fachbegriff: Glutäago) mit chronisch überlasteten Muskeln und Bändern am Becken zusammenhängen, scheint einleuchtend: Sie müssen beim Sitzen das Kreuz aufrecht halten. Und wer sitzt schon achtzig Prozent eines Achtstundentages oder länger ergonomisch korrekt?*

***URL: https://www.onmeda.de/forum/krebserkrankungen/2884262-blasenkrebs***

*Ich bin männlich und 22 . War heute beim Hausarzt Blut +++ wurde festgestellt und Leukos ++. Angefangen hat alles vor 4 Wochen mit Ziehen im Bauch danach in Leiste und anschließend nur bei Bewegungen und wenn ich eine enge Hose an hatte. Bin seit 7 Jahren jährlich beim Urologen hatte in 3 Fällen einmal Blut im Urin bei der nächsten Untersuchung war es weg; nun mache ich mir sorgen wegen einem Tumor ; derzeit nach dem ich auf Toilette war teilweise ein leichtes druckgefühl in Blasengegend. Auch früher war das Blut nur unterm Mikroskop sichtbar ; heute waren keine Bakterien im Urin was ja eine Entzündung ausschließt. bitte um Ratschläge außer geh zum Urulogen und ich hab wahnsinnige Angst vor einer Blasenspiegelung.*

Die nachfolgenden Textausschnitte enthalten keine „Gesundheitsinformationen“:

*URL: https://www.netzkatzen.de/threads/verdacht-auf-gesaeugetumor.170321/*

*Letzten Sonntag entdeckte ich einen Knubbel an der Zitze, Montag waren wir beim TA und da wurde noch ein zweiter Knubbel bzw. Zyste auf der anderen Seite entdeckt :(. die eine Zyste ging auch auf und Flüssigkeit trat heraus. Es wurde Blut abgenommen und jetzt sitze ich hier und warte auf den Anruf vom TA dann geht das Prozedere weiter mit Röntgen etc. ich habe mich natürlich eingelesen und bin sehr traurig und habe Angst um meine Miezi. Das warten macht mich wahnsinnig. Falls eine OP nötig und möglich werde ich das bei meiner TA des vertrauens machen lassen aber erst brauch ich die verdammten Ergebnisse*

***URL: https://www.garnelio.de/krebse***

*Wer sich mit Aquarienkrebsen beschäftigt, wird sehr schnell merken das diese sanften riesen nicht nur gut aussehen sondern auch interessant zu beobachten sind. In der Rubrik Krebse finden sie farbenprächtige Flusskrebse der Gattung Cherax. Wunderschöne nordamerikanische Krebse der Gattung Procambarus die es mittlerweile in vielen Farbformen gibt und die Zwerge unter den Flusskrebsen die Zwergflusskrebse der Gattung Cambarellus angehören und nur wenige cm groß werden.*

***URL: http://www.spiegel.de/panorama/nachwehen-boxerin-bricht-raab-die-nase-a-124978.html***

*7,65 Millionen Menschen hatten vor dem Fernseher live zugesehen, als Raab am vergangenen Donnerstag im Kölner "Capitol" jede Menge Prügel im Kampf gegen die zierliche Sportlerin einstecken musste. In der dritten von fünf Runden verpasste die 1,60 Meter kleine und 51 Kilogramm leichte Weltmeisterin dem Moderator eine blutige Nase. "Im Fernsehen sieht das viel schlimmer aus, als es war", hatte Raab zunächst beschwichtigt. Am nächsten Morgen aber bildete sich einem Bericht der "Bild" zufolge ein so genanntes Brillenhämatom. Das Nasenbein sei glatt durchgebrochen, habe der Arzt diagnostiziert .Schon 1997 hatte der Frankfurter Rapper und Musikproduzent Moses Pelham Raab im Streit die Nase zertrümmert. 10.000 Mark Schmerzensgeld kassierte Raab damals. Er musste operiert werden und war vier Wochen lang arbeitsunfähig. Auch der jüngste Nasenbeinbruch hat Folgen: Mit der Boxerei sei es nun erst mal vorbei, hieß es.*
